# The Influence of the Amazon Echo Dot on Loneliness Among Older Adults Living Alone in Underserved Communities

**DOI:** 10.1093/geroni/igaf122.2491

**Published:** 2025-12-31

**Authors:** Rie Suzuki, Charlotte Tang, Jennifer Blackwood, Brent Gasser, Ashton Herrick, Annaliese Elliott

**Affiliations:** University of Michigan-Flint, Flint, Michigan, United States; University of Michigan-Flint, Flint, Michigan, United States; University of Michigan-Flint, Flint, Michigan, United States; University of Michigan-Flint, Flint, Michigan, United States; University of Michigan-Flint, Flint, Michigan, United States; University of Michigan-Flint, Flint, Michigan, United States

## Abstract

Loneliness is associated with a higher mortality rate in older adults. Virtual home assistants (VHAs) offer potential benefits for social engagement, but their specific impact on loneliness among older adults in underserved communities is not well documented. The purpose of this pilot study was to examine the effects of VHA use on loneliness in older adults living alone. A pre-post mixed methods study was conducted from 2024 to 2025 with participants from four senior centers in the Greater Flint Area. Inclusion criteria were being 60 years or older, living alone, self-reported isolation, smartphone use, Wi-Fi access, no prior VHA use, and a modified cognitive status telephone interview with a score of 21 or higher. Participants received an Echo Dot, a smart light, and $100 for participating in the study, with eight-week weekly follow-ups. Of the 13 who completed the study, most were white, single (divorced or widowed), female, and had some college education. At baseline, 54% of participants were dissatisfied with AI technology. In the semi-structured follow-up interview, most stated that the device had not significantly reduced loneliness in their daily lives. However, some appreciated features such as music and interaction and commented on their value to society. Others acknowledged the benefits of the device in alleviating loneliness and commented that they found it enjoyable to be ‘present’ and receive advice. The positive feedback from participants and belief in the future use of feasible VHAs suggest that they have the potential to tackle loneliness among older adults in underserved communities.

